# Supplementary material for: Inhibition of the YAP-MMB interaction and targeting NEK2 as potential therapeutic strategies for YAP-driven cancers
Source: Oncogene. 2024 Jan 5;43(8):578–93. doi: 10.1038/s41388-023-02926-w (PMC10873197; doi:10.1038/s41388-023-02926-w)
Supplement: Supplementary file 1 — Supplemental Material [file 41388_2023_2926_MOESM1_ESM.pdf]

Inhibition of the YAP-MMB interaction and targeting NEK2 as potential therapeutic strategies for YAP-driven cancers

Marco Jessen, Dörthe Gertzmann, Franziska Liss, Franziska Zenk, Laura Bähner, Victoria Schöffler, Clemens Schulte, Hans Michael Maric, Carsten P. Ade, Björn von Eyss and Stefan Gaubatz

SUPPLEMENTAL MATERIAL

SUPPLEMENTAL FIGURES AND TABLE LEGENDS

Supplemental Figure S1: Nuclear localization of YAP in HeLa cells

HeLa cells were immunostained for YAP revealing nuclear localization. Scale bar: 25 µm.

Supplemental Figure S2: MY-COMP leads to errors in cell division

A) Scheme of the constructs used B) The MY-COMP constructs shown in A were expressed in HeLa cells and FACS was performed to determine the percentage of in different phases of the cell cycle. GFP-positive cells were analyzed. n=3. Statistical significance of differences in polyploid cells calculated by ordinary one way ANOVA. *** < 0.001. ns: not significant.

Supplemental Figure S3: MY-COMP inhibits YAP mediated transcription of MMB-target genes A) Immunoblot analysis of cytoplasmic (C) and nuclear (N) lysates of MCF10A cells expressing ER-YAP2SA treated with 4-OHT for 14 hours or left untreated. Histone H2B served as a nuclear marker and tubulin as cytoplasmic marker. B) and C) RT-qPCR and immunoblot analyses of MCF10A ER-YAP2SA cells treated with 4-OHT for the indicated times (n=2 replicates). Actin served as a control for the immunoblot. D) MY-COMP was stably expressed in MCF10A-ER-YAP2SA cells. Cells were treated with or without doxycycline and the

expression of MY-COMP was analyzed by immunostaining with an HA-antibody. Scale bar: 25 µm. E) and F) MCF10A cells stably expressing ER-YAP5SA and doxycycline-inducible MY-COMP were treated with 4-OHT and doxycycline as indicated. E) Immunoblotting was used to analyze the expression of the indicated proteins. Actin served as a control. F) RT-qPCR to analyze the expression of the *CYR61* mRNA. Error bars indicate s.d. of three biological replicates.

Supplemental Figure S4: Expression of MY-COMP in melanoma cell lines.
The indicated uveal melanoma cell lines and cutaneous melanoma cell lines were stably transfected with doxycycline inducible MY-COMP. Dose dependent expression of MY-COMP constructs was verified by immunoblotting with an HA-antibody. Tubulin served as a control.

Supplemental Figure S5: MMB-target genes regulation by MY-COMP and YAP
GSE analysis of MMB-target gene signature upon MY-COMP expression or YAP/TAZ depletion in 92.1 cells.

Supplemental Figure S6: shRNA mediated depletion of NEK2 inhibits the growth of uveal melanoma cell lines. A) Western blot of NEK2 expression after siRNA-mediated depletion of YAP/TAZ. * denotes a background band that is visible in some NEK2 immunoblots. B) Schematic illustration of the lentiviral pInducer10 construct used to express the shRNA targeting NEK2 and turboRFP(tRFP). C) UM cell lines with inducible constructs for shRNAs against NEK2 or a luciferase (shctrl) as described in B) were generated. Cell lines were treated with 1µg/ml doxycycline for three days and *NEK2* expression relative to untreated control cells was analyzed by RT-qPCR. *GAPDH* expression was used for normalization. Error bars show SD of three independent experiments. D) NEK2 expression of cell lines described in B was analyzed by immunoblot. Vinculin served as a control. E) UM and CM cells were seeded at low density and left treated or untreated with the indicated concentrations of doxycycline for 10 days. Cells were fixed and stained with crystal violet. Plots show

quantification of crystal violet staining. N=3 biological replicates each performed in triplicates. Error bars indicate SEM of one representative experiment.

Supplemental Figure S7: Depletion of NEK2 in uveal melanoma cells results in apoptosis. UM and CM cells were treated with the indicated concentrations of INH1 for 4 days. Cell cycle phases were determined by PI staining followed by FACS analysis. See Figure 8E for a quantification of cells in subG1.

Supplemental Table 1

List of primer sequences and siRNAs

Supplemental Table 2

List of antibodies

Supplemental Table 3

Expression changes upon ER-YAP2SA and MY-COMP expression in MCF10A cells.

Related to Figure 3.

Supplemental Table 4

Fold changes and q-values of MMB target genes upon expression of MY-COMP in 92.1 cells. Related to Figure 6A.

Supplemental Table 5

Fold changes and q-values of MMB target genes upon depletion of YAP in 92.1 cells. Related to Figure 6B.

Supplemental Table 6

Significance for association with overall survival (OS) of uveal melanoma and skin cutaneous melanoma patients based on stratifying for expression quintiles of YAP/MMB target genes. Related to Figure 7A.

Supplemental Figure S1

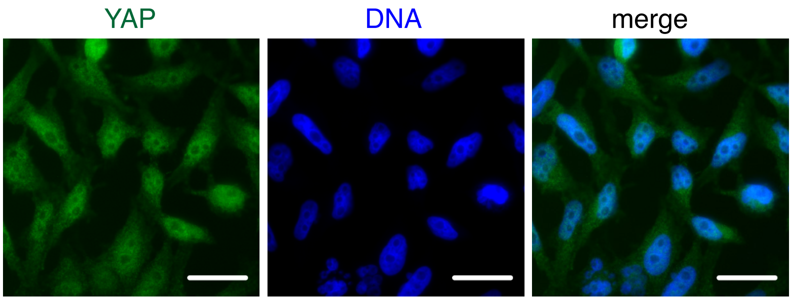

Supplemental Figure S2

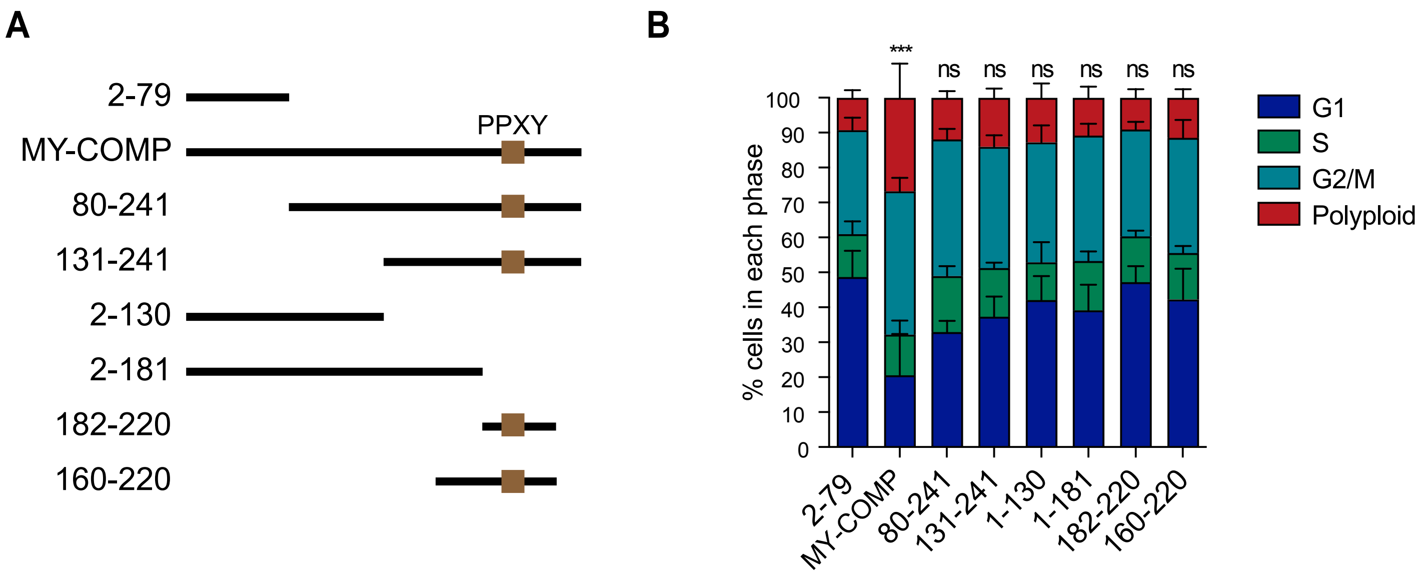

Supplemental Figure S3

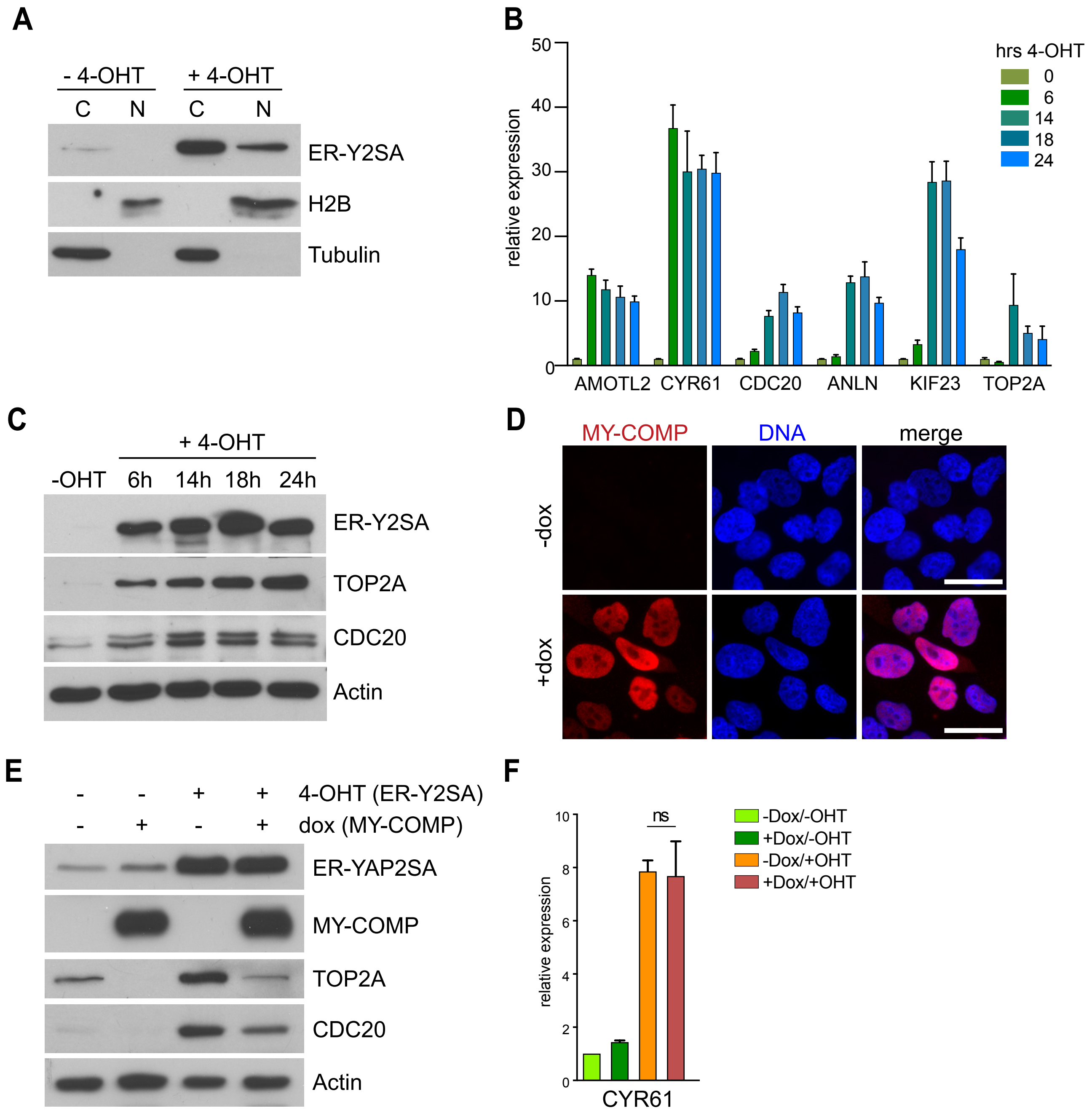

Supplemental Figure S4

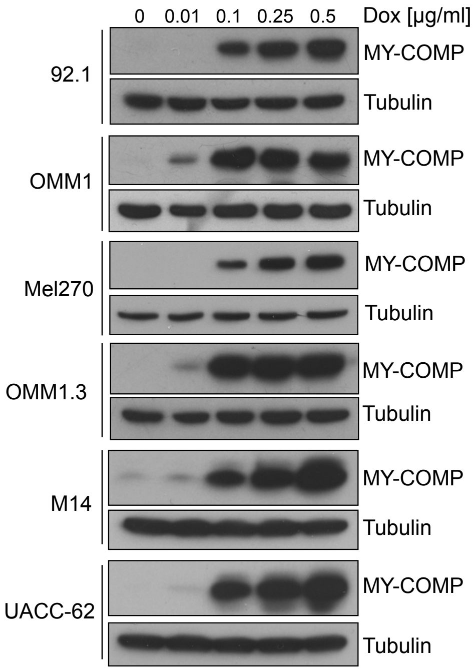

Supplemental Figure S5

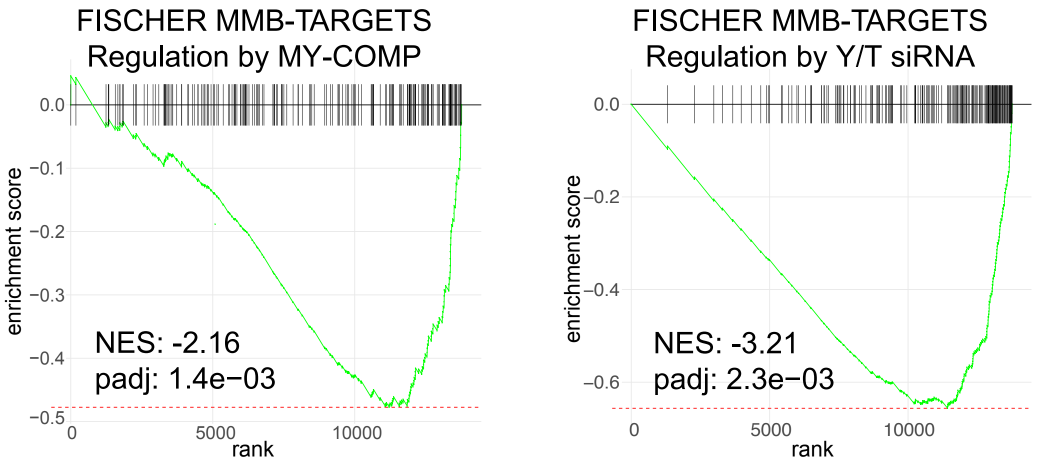

Supplemental Figure S6

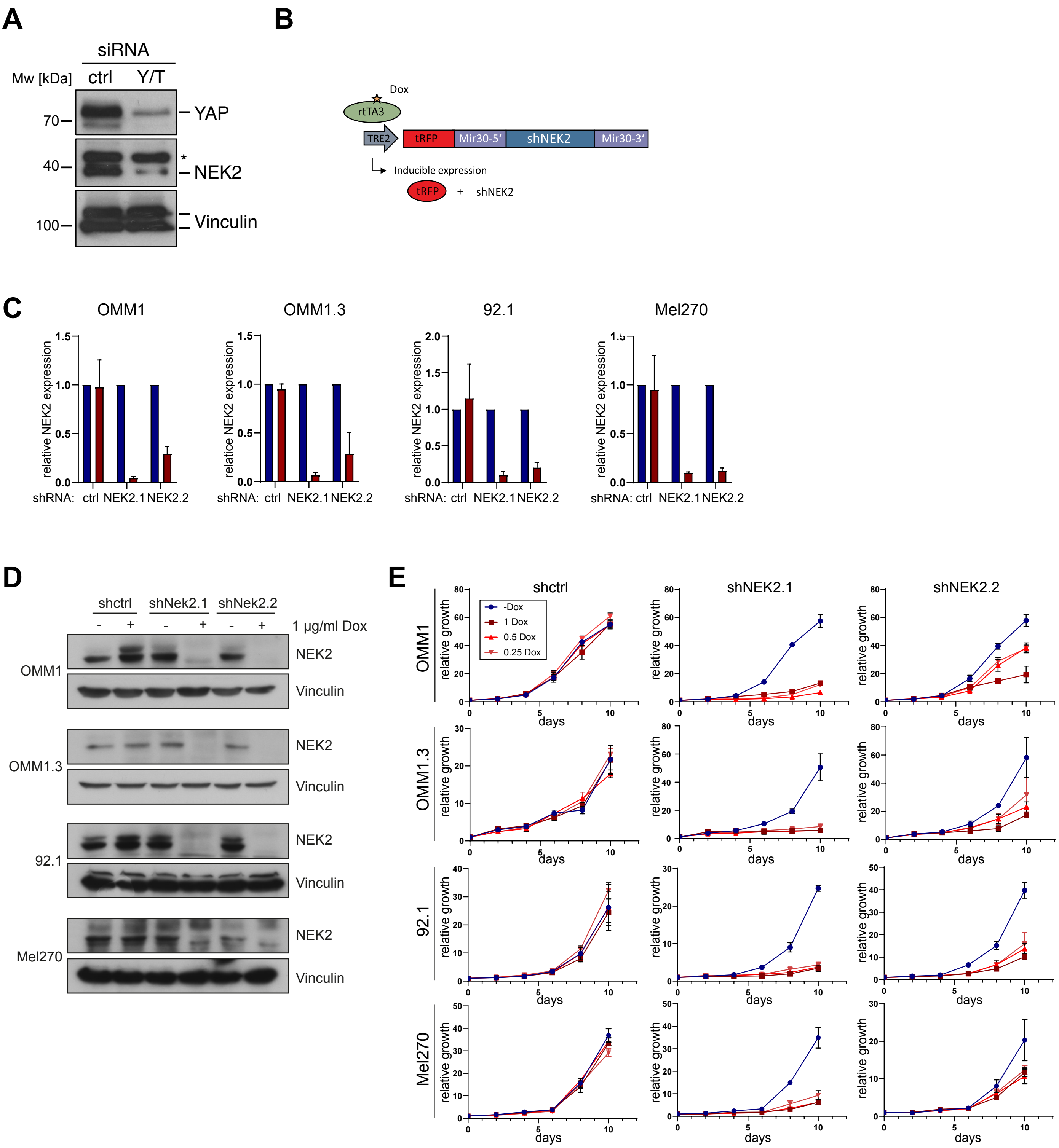

Supplemental Figure S7

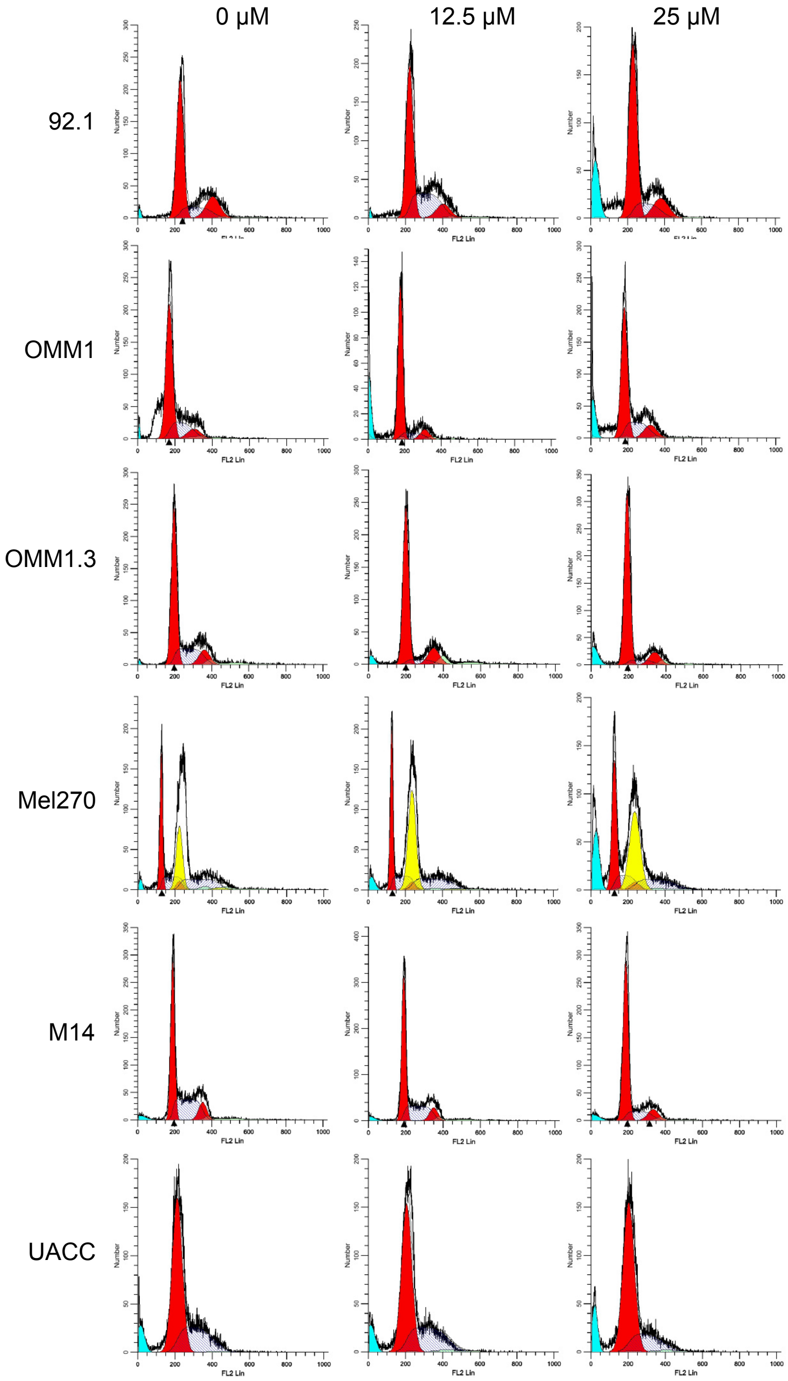

Supplemental Table 1: Oligonucleotide and siRNA sequences

RT-qPCR primers

Gene	fw primer	bw primer
AMOTL1	AGGCTGCAGAGAGACAATGAG	CTCAGAGAGCCGCTGGATT
ANLN	GCGAGCTAGACAGCCACTTT	TTTTTGATGGCGATGGTTTT
ARHGAP11A	CAGAACACCTTCTATTACACCTCAAG	GCATTTGGTGTAAGAATCACTGG
CYR61	CAACCCTTTACAAGGCCAGA	TGGTCTTGCTGCATTTCTTG
DEPDC1	CCTATGGAGAGTCAGGGTGTG	CGAAAAGATGTGGTAACTTCATTC
GAPDH	GCCCAATACGACCAAATCC	AGCCACATCGCTCAGACAC
KIF23	CCTAACGTCCCGCAGTCTT	AGGTTTCCGGGGTGTCTTAG
KIF4A	TGGTCAGACAGCCCAGATG	TCTTCTAGCTTGGCGTTCATT
NCAPH	ACCTCAAACCAGGCACCA	TCTTCATAATGCTCAGTCTCTACCC
NEK2	CATTGGCACAGGCTCCTAC	TCATGGAGCCATAGTCAAGTTCT
PLK1	AAGATCTGGAGGTGAAAATAGGG	AGGAGTCCCACACAGGGTCT
TAZ	GTATCCCAGCCAAATCTCGT	TTCTGCTGGCTCAGGGTACT
TOP2A	TCTGGTCCTGAAGATGATGCT	TTAGTTAACCATTCCTTTCGATCA
YAP	GACATCTTCTGGTCAGAGATACTTCTT	GGGGCTGTGACGTTCATC

3C-qPCR

Fragment number	sequence
anchor	GCCCTCTCAGGCACAAGAAAGG
1	GCCGTTTGTCATCTCTCACTGGG
2	GACTCTGGCCCTGAAGGAATGC
3	CCTAGAGCTGGGGCATTTGGC
4	GCTCAGGCAGTACTGCTGGTC
5	TACATGCCTGCAGCCCTCCTG
6	TGAGCCGAGATCATGCCACTGC
7	GGTTGTGGCACTGGAGACAGATTC
8	CGCGGAAGTGCAGAGAAGCAG
9	GGTCAAGCGCTGCAGAGATGC
10	GAGGCTTCAGGGCCATGATGAGG
11	GTCCCTAACCCCACACAGTCAGAC
12	CCCTCCAGGGTGACAAAGGGAC
loading control fw	GTCGCTTGAGCCCAGGAGTTC
loading control bw	CTCTTGGCCTCGAGCAATCCG

siRNAs

Gene	Name/ sequence	Reference
ctrl	Cat#4390843	Thermo Fisher Scientific
YAP	UCUCUGACCAGAAGAUGUC	Azzolin Cell 2014; 158:157–70.
TAZ	ACGUUGACUUAGGAACUUU	Azzolin Cell 2014; 158:157–70.
B-MYB	GAAACGAGCCUGCCUUACAUU	Schmit Cell Cycle 2007; 6:15, 1903-1913
LIN9	GGAAGAGAGAUCAGCAUUAUU	Schmit Cell Cycle 2007; 6:15, 1903-1913

Supplemental Table 2: Antibodies used in the study

Protein	host species	Source	Identifier	Application
alpha-tubulin	mouse	Sigma	T6074; RRID: AB_477582	IF
anti-β-Actin	mouse	Santa Cruz Biotechnology	Cat# sc-47778; RRID: AB_626632	WB
B-MYB	mouse	-	clone LX015.1; RRID: not available	WB
CDC20	mouse	Santa Cruz Biotechnology	Cat# sc-13162; RRID: AB_628089	WB
flag (M2)	mouse	Sigma	Cat# F3165; RRID: AB_259529	WB
gamma-tubulin	rabbit	Sigma	T5192; RRID: AB_264690	IF
HA (HA.11)	mouse	HISS	Cat# MMS-101P; RRID: AB_2314672	WB
Histone H2B	rabbit	Abcam	Cat# ab1790, RRID:AB_302612	WB
Histone H3 (acetyl K27)	rabbit	Merck	Cat# 07-360; RRID: AB_310550	CUT&RUN
Histone H3 (mono methyl K4)	rabbit	Abcam	Cat# ab8895; RRID: AB_306847	CUT&RUN
Histone H3 (tri methyl K4)	rabbit	Abcam	Cat# ab8580; RRID: AB_306649	CUT&RUN
Histone H4 (acetyl K5,8,12,16)	rabbit	Abcam	#06-598	CUT&RUN
IgG	rabbit	Sigma	Cat# I5006; RRID: AB_1163659	CUT&RUN
IgG	mouse	Sigma	Cat#I5381;RRID:AB_1163670	CUT&RUN
LIN9	rabbit	Bethyl	Cat# A300-BL2981; RRID: N/A	WB
NEK2	mouse	BD Biosciences	610593; RRID: AB_397933	WB
phospho-YAP (S127)	rabbit	Cell Signaling	Cat# 4911; RRID: AB_2218913	WB
TOP2A	mouse	Santa Cruz Biotechnology	Cat# sc-365916; RRID: AB_10842059	WB
Vinculin	mouse	Sigma	V9131; RRID: AB_477629	WB
YAP	mouse	Santa Cruz Biotechnology	Cat# sc-101199;RRID: AB_1131430	WB
YAP	rabbit	Cell Signaling	Cat# 14074; RRID: AB_2650491	IHC
YAP	rabbit	Novus Biologicals	NB110-58358 ; RRID:AB_922796	CUT&RUN
Anti-mouse HRP conjugated		GE Healthcare	Cat# NXA931; RRID: AB_772209	WB

Supplemental Table 6

gene	p-value UVM	p-value (SKCM)
NEDD9	0.000208753	0.037072884
CDC25B	0.000588943	0.441650859
KIF20A	0.001794255	0.033327048
RRM2	0.005048059	0.411319142
SKA1	0.009665771	0.163408569
CDC25C	0.010509242	0.078117992
ASF1B	0.010721858	0.044023866
TPX2	0.022868364	0.270745245
H2AFV	0.027098992	0.116804935
NEK2	0.028567756	0.650056761
IQGAP3	0.031032975	0.001684824
STIL	0.035435754	0.727552757
NUSAP1	0.038886372	0.98435199
CKS1B	0.046319613	0.17627508
KIF18B	0.050078456	0.169090775
CHEK2	0.050956835	0.321538962
AURKB	0.055967001	0.006794848
CCNA2	0.064948158	0.218099316
RAD21	0.086082933	0.025857304
CENPA	0.101541651	0.031993769
RACGAP1	0.114566307	0.452055672
PIM1	0.121138151	0.040263734
KIF2C	0.148341343	0.012025081
C15ORF23	0.199719692	0.61005831
DBF4B	0.241450035	0.06709812
NCAPD2	0.250878195	0.203874428
CENPN	0.269690208	0.6310422
FAM102B	0.285514438	0.16497173
INCENP	0.329642012	0.098372102
BRD8	0.419880382	0.098032762
ESPL1	0.438853653	0.003614506
DCAF16	0.628284186	0.375714613
RAD54L	0.828725955	0.147890486
